# Electrical Stimulation of the Ear, Head, Cranial Nerve, or Cortex for the Treatment of Tinnitus: A Scoping Review

**DOI:** 10.1155/2016/5130503

**Published:** 2016-06-15

**Authors:** Derek J. Hoare, Peyman Adjamian, Magdalena Sereda

**Affiliations:** ^1^NIHR Nottingham Hearing Biomedical Research Unit, Otology and Hearing Group, Division of Clinical Neuroscience, School of Medicine, University of Nottingham, Nottingham NG1 5DU, UK; ^2^MRC Institute of Hearing Research, University Park, Nottingham NG7 2RD, UK

## Abstract

Tinnitus is defined as the perception of sound in the absence of an external source. It is often associated with hearing loss and is thought to result from abnormal neural activity at some point or points in the auditory pathway, which is incorrectly interpreted by the brain as an actual sound. Neurostimulation therapies therefore, which interfere on some level with that abnormal activity, are a logical approach to treatment. For tinnitus, where the pathological neuronal activity might be associated with auditory and other areas of the brain, interventions using electromagnetic, electrical, or acoustic stimuli separately, or paired electrical and acoustic stimuli, have been proposed as treatments. Neurostimulation therapies should modulate neural activity to deliver a permanent reduction in tinnitus percept by driving the neuroplastic changes necessary to interrupt abnormal levels of oscillatory cortical activity and restore typical levels of activity. This change in activity should alter or interrupt the tinnitus percept (reduction or extinction) making it less bothersome. Here we review developments in therapies involving electrical stimulation of the ear, head, cranial nerve, or cortex in the treatment of tinnitus which demonstrably, or are hypothesised to, interrupt pathological neuronal activity in the cortex associated with tinnitus.

## 1. Introduction

Tinnitus affects about 16% of the adult population [1, 2] and is strongly associated with hearing loss [3]. There is as yet no cure and so clinical management typically involved education and sound-based therapies [4]. It is associated with pathological neural activity in the central auditory system, possibly resulting from suboptimal or maladaptive response to a peripheral lesion (hearing loss) within a certain frequency range resulting from ageing [5] or noise exposure [6]. While the loss of cochlear hair cells causes elevated thresholds in that frequency range, at the same time spontaneous activity of several auditory structures can be enhanced. Increased spontaneous firing rate and spike-timing dependent plasticity facilitate increased synchrony of firing within and across neural assemblies; both hyperactivity and increased synchrony are proposed as neural correlates of tinnitus [7, 8]. Although it is difficult to separate contributions of the two mechanisms based solely on experimental data [9], a recent review of physiological evidence suggests that changes in spontaneous firing alone are unlikely to generate a tinnitus percept [10].

In the intact brain, transient synchronization of neuronal oscillatory activity is thought to be the mechanism that facilitates functional connectivity between different regions, which binds the activity from distributed neural ensembles into a coherent representation of cognitive and sensory functions [11]. Conversely, altered synchronization has been observed in a number of psychiatric and cognitive conditions, including schizophrenia [12], Alzheimer's disease [13], and attentional deficits [14]. Likewise, in tinnitus, a number of studies have reported tinnitus-related functional connectivity between disparate regions of the brain based on brain oscillations; for example, connectivity between the anterior cingulate cortex (ACC) and both the right frontal lobe and right parietal lobe strongly correlates with tinnitus distress [15–17]. Studies typically attempt to correlate brain oscillations and their interregional statistical interdependencies with observed changes in perception and behaviour. More recently, a number of studies take an alternative approach in that they attempt to alter brain oscillations by means of brain stimulation to observe changes in behaviour. This is based on the assumption that modulating brain rhythms may affect cognitive performance and thus can be used to treat neurological disorders. Numerous studies over many decades indicate that low-intensity electrical currents can modulate network dynamics noninvasively in humans. Electrical stimulation has also been applied invasively in humans, either on the cortical surface or as deep implanted electrodes [18]. However, the precise neural mechanism by which changes occur at both local and network levels is not fully understood (for a review of the animal literature see Reato et al. [19]). It is not clear how the effects at the single neuronal level influence the network activity both spatially and temporally. Moreover, with regard to noninvasive techniques, it is not known which precise location of the brain is being stimulated. Nevertheless, studies report changes in perception during or following electrical stimulation of the brain.

To date, a small number of neurostimulation techniques for tinnitus have been the subject of either a systematic review and meta-analysis (transcranial Direct Current Stimulation (tDCS) [20], cochlear implantation [21, 22]) or a scoping review (tDCS [23]). Here the objective was to scope the literature on approaches to electrical stimulation of the ear, head, cranial nerve, or cortex for tinnitus which are more experimental insofar as they have not yet been the subject of a scoping or systematic review.

## 2. Methods

All study types were included in this scoping review. Inclusion was determined according to the implementation of an intervention of interest and the measurement of an outcome of interest. Interventions included in this review used noninvasive electrical stimulation of the ear, transcranial alternating current stimulation (tACS), vagal nerve stimulation (VNS), transcutaneous vagal nerve stimulation (tVNS), procedures involving direct cortical stimulation, or paired electrical and acoustic stimulation. In terms of outcomes, of interest was whether the intervention had any potential clinical effect on symptom severity, measured as the global score on a self-report measure of tinnitus, generalised anxiety, depression, or quality of life, and equally whether there were adverse effects of the intervention. Our second interest was the potential neurophysiological mechanisms of any effects, for example, change in neurophysiological function as measured by magnetoencephalography (MEG) or electroencephalography (EEG).

### 2.1. Search Strategy

Studies were identified from the results of electronic database searches. Databases were PubMed, Web of Science, and Google Scholar. Database searches were conducted in December 2015 using the search terms Tinnitus, OR tinnit^*∗*^, AND (Transcutaneous Electric Nerve Stimulation) OR (Vagus Nerve Stimulation) OR (Electric Stimulation) OR (Electric Stimulation Therapy) OR (tACS or VNS or tVNS) OR (neuromodulat^*∗*^ or neurostim^*∗*^) OR (vagus or vagal or transcutaneous or transcranial or Transdermal or Percutaneous or cutaneous or AC near stimul^*∗*^) OR (alternating near current near stimul^*∗*^) OR (Electrostimul^*∗*^ or neurostimulation or electro-stimul^*∗*^ or neuro-stimul^*∗*^ or “electro stimul^*∗*^” or neuro stimul^*∗*^) OR (electrical stimul^*∗*^ or electric stimul^*∗*^).

To identify ongoing or unpublished trials we searched ClinicalTrials.gov and conducted a hand search for recently published study protocols relevant to this review.

## 3. Results and Discussion

From initial 642 search records, 46 studies were selected for full-text review by DH. All authors then independently selected relevant studies according to our inclusion criteria. Nineteen human studies met our criteria for inclusion and are reviewed here by class of intervention. These included three studies using noninvasive electrical stimulation of the ear (reported 2013-2014), two studies of transcranial alternating current stimulation (tACS) (reported 2013-2014), one pilot study and one case study using vagal nerve stimulation (VNS) (reported 2014-2015), five studies using transcutaneous vagal nerve stimulation (tVNS) (reported 2012–2015), one study examining vestibulocochlear nerve stimulation (reported in 2007), and seven studies involving different forms of direct cortical stimulation (reported 2004–2015). A summary of findings from clinical studies is given in Table 1.

### 3.1. Noninvasive Electrical Stimulation of the Ear

Suppression of tinnitus by electrical stimulation of the preauricular skin, mastoid, eardrum, promontory, and round window and within the cochlea has been employed for many years [24, 25]. Efficacy of the electrical stimulation reported by some early studies varies from 7 to 82% of patients perceiving benefit for their tinnitus [24–35]. A more recent study by Mielczarek et al. [36] assessed the effectiveness of electrical stimulation of the hearing organ on tinnitus (severity not measured) in patients with cervical spine degeneration, also examining any additive effect of cervical spine kinesitherapy (therapy involving muscle movement) on tinnitus. The frequency of the stimulation, delivered through the electrode placed in the ear canal targeting the cochlea, was adapted according to tinnitus frequency. The treatment involved 15 applications of 4 min electrical stimulation 3-4 times a week. After the treatment in both groups the number of ears (location not specified) with permanent tinnitus decreased from 88% in the group with electrical stimulation only and 90% in the group with combined electrical stimulation and kinesitherapy to 43 and 54%, respectively. In 37% of ears after electrical stimulation and 28% of ears after combined treatment tinnitus disappeared completely. In addition self-reported and audiometric improvement of hearing was noted in both groups, in 34% and 26% of ears, respectively. This study was followed by a double-blind placebo-controlled study in 120 patients using the same method of electrical stimulation of the ear [37]. The placebo involved application of the electrode to the ear canal without any current. Similar to their earlier study, proportion of ears with permanent tinnitus decreased from 89% before treatment to 49% immediately after treatment. In 34% of ears tinnitus disappeared completely. Interestingly, in the placebo group, the proportion of ears with tinnitus also decreased immediately after the control from 86% to 71%. Tinnitus disappeared completely in four ears in the control group. However, the effect was reported as stable in the treatment group after 30 and 90 days; the placebo effect was not maintained. Moreover, in the treatment group, there was a statistically significant improvement in audiometric thresholds, with patients also reporting subjective improvement in 30% of ears. There was no deterioration of hearing after the treatment. Whilst impressive, tinnitus was not measured in these studies using a multi-item tinnitus questionnaire so the results have to be considered with caution.

Lee et al. [38] investigated effectiveness of transcutaneous electrical nerve stimulation (TENS) applied to the external pinna in 65 participants with moderate severity tinnitus (randomized such that 45 were in the treatment arm and 20 received sham stimulation). Participants received TENS treatment twice a week for four weeks. Only two participants had a long-term improvement after treatment with TENS (effects maintained at three months) and it was noted that both had low frequency tinnitus and milder hearing losses. It would be interesting to evaluate TENS in a group with these characteristics. No studies of TENS for tinnitus were identified as ongoing, and a systematic review is not indicated at present.

### 3.2. Transcranial Alternating Current Stimulation (tACS)

tACS involves the delivery of alternating current (constant polarity changes) between electrodes placed on the skin over cortical regions of interest. It is hypothesised to affect upregulation and downregulation of synapses and may have an effect on oscillatory cortical activity, indicating it for tinnitus [39]. In the first study of its kind Vanneste et al. [40] compared single session tACS, tDCS, and transcranial random noise stimulation (tRNS; equating to tACS modified to have a 0 mA offset applied at random frequencies; frequencies ranged from 0.1 to 100 Hz). One hundred and eleven tinnitus patients were randomly assigned to the three study arms. The main outcomes were tinnitus loudness and annoyance, measured before and after treatment on a 10-point numeric rating scale. Only the group receiving tRNS showed significant within and between group effects of the intervention, on both rating scales, with the authors concluding this to be the most clinically effective for transient tinnitus suppression within a single session. It was suggested that the lack of an effect of tACS might have been due to the rather weak current used (1.5 mA for 20 min), a consideration for future studies.

Vanneste et al. [41] also compared tinnitus suppression using single sessions of individual alpha-modulated tACS or tDCS in a randomized placebo-controlled study. Fifty participants were randomly allocated to receive either real stimulation (up to 10 mA and lasting 20 minutes) or sham stimulation (current was turned off after 30 seconds). Again, the main outcomes were tinnitus loudness and annoyance, measured on a 10-point numeric rating scale. EEG was also recorded immediately after treatment for those receiving real tACS. The authors reported a positive effect of tDCS ratings of tinnitus loudness or annoyance but no significant effect of tACS compared to sham stimulation. EEG analyses also showed no effect on power between pre- and post-tACS.

Most recently, in a retrospective analysis of 228 patients who received therapy for tinnitus, Claes et al. [42] examined the effects of single or repeated sessions of alpha-modulated tACS (ramped to 2.0 mA) and tRNS as described earlier. Effects on tinnitus loudness and annoyance were measured using simple 10-point numeric rating scales. In both interventions stimulation was applied to the auditory cortex in sessions lasting 20 minutes. In this study, tACS had no effect on tinnitus. tRNS however was associated with reduced tinnitus loudness, both within a single session and additively across sessions. tRNS was also associated with an improvement after a single session; multiple sessions had no additive effect. Physiological effects were not measured.

The study of tACS is therefore at an early stage, although there are no strong indicators for further study, and no studies were identified as ongoing. However, the studies reported here were all conducted by the same research group. Current protocols need replication to discount them, and alternative protocols should explore the optimal conditions of tACS and whether any subgroups of tinnitus patient show benefit. As discussed by Claes et al. [42], potential influencing variables including age, sex, and tinnitus characteristics need to be considered as variables in future work.

tRNS is the subject of at least two ongoing studies. Langguth and colleagues are conducting a safety/feasibility/efficacy study (NCT01965028) with 30 participants, all receiving high-frequency tRNS daily for two weeks, with a primary outcome of number of participants with a ≥5-point tinnitus questionnaire (TQ [43]) score at long-term follow-up. This contrasts with previous studies where only acute effects were measured and simple rating scales as opposed to clinical questionnaire tools were used. The same group are also examining the safety/feasibility/efficacy of daily low frequency tRNS (NCT02615600) using the same protocol.

### 3.3. Vagal Nerve Stimulation (VNS): General Concept and Animal Studies

One of the postulated ways of reversing the pathological plastic changes in the auditory system associated with hearing loss and tinnitus is through targeted auditory stimulation or auditory discrimination training [44]. However, to date, the clinical benefit of auditory stimulation approaches to treating tinnitus has been limited [45] and only temporary relief has been demonstrated. It is postulated that auditory stimulation alone is not enough to affect plastic changes in the brain and so research attention has shifted to the forebrain cholinergic and noradrenergic systems that play a significant role in modulating cortical plasticity. Studies in animals looking at the electrical stimulation of the cholinergic nucleus basalis provided evidence for pronounced and long-lasting changes in cortical reorganization [46] and led to the idea that pairing auditory stimulation with electrical stimulation could be used to treat tinnitus [47]. However, stimulation of the nucleus basalis is an invasive procedure and therefore not a viable option for widespread use in tinnitus patients.

In 2011, Engineer and colleagues published a study looking to obtain similar effect to nucleus basalis stimulation but using much less invasive electrical stimulation of the vagus nerve (10th cranial nerve; VNS) in noise-exposed rats [48]. The VNS approach was considered promising as previous clinical studies showed it can alleviate epilepsy [49] and depression [50]; it is now a Food and Drug Administration approved treatment for drug-resistant epilepsy and depression. Engineer et al. [48] used adult rats with noise-induced hearing loss presumed to have a related tinnitus in the region of 8–10 kHz as verified with the startle reflex paradigm [51]. Noise-exposed rats showed a significant cortical map distortion, decreased frequency selectivity, increased tone-evoked response, and increased spontaneous neural activity in the auditory cortex and increased cortical neural synchrony. A regime consisting of acoustic stimulation composed of multiple tones at frequencies outside the tinnitus region, paired with VNS, was associated with reversal of both behavioural and physiological markers of tinnitus, except for increased spontaneous activity which was still observed. Neither acoustic stimulation alone nor VNS alone led to the same changes. The authors postulated that while acoustic stimuli are essential to activating particular neural populations within the auditory cortex (to be able to target neuroplastic change), the VNS component of the treatment was essential to promoting those plastic changes through what Engineer et al. [48] term as “synergistic action of multiple neuromodulators.” Specifically, it was postulated that acetylcholine and muscarinic receptors are involved in VNS mechanism of action [48, 52].

### 3.4. Human Studies Using Implanted VNS

Several studies explored the safety and efficacy of the VNS paired with acoustic stimulation in humans. De Ridder and colleagues [53] conducted a pilot study with 10 participants with chronic moderate-to-catastrophic tinnitus severity tinnitus. Participants received 2.5-hour treatment for 20 days. The patients heard tones, excluding the tinnitus-matched frequency, paired with brief electrical stimulation of the vagus nerve. Both clinical and physiological (electroencephalography, EEG) effects of VNS were reported. Whole group analysis revealed a decrease in Tinnitus Handicap Inventory (THI [54]) score of 11% immediately after the treatment and 12% at four-week follow-up relative to baseline (not a clinically significant score change). Exploratory analyses suggested that taking psychoactive medications such as antidepressants may inhibit the benefit to be had from the treatment. The mean decrease in THI scores for nondrug group was 29% immediately after treatment and 26% at follow-up, while for the drug group the change was minimal. Authors also reported a more pronounced reduction of Minimum Masking Levels (MMLs) in the nondrug group (18.8 dB reduction) than in the drug group (4.3 dB reduction). From EGG recordings (performed in 7 participants only due to technical problems) they concluded that VNS decreased band power in the delta (1–4 Hz) and theta (4–8 Hz) bands in subjects who responded to therapy, with increased band power in those bands observed in subjects who did not respond to the therapy. For both delta and theta bands the difference in band power change between VNS and sham trials strongly correlated with their THI scores (*r* ≥ 0.74). There are several caveats about the results of that study however. Only one participant had a clinically significant improvement (20 or more points) on the THI, 6 had an improvement that was not clinically meaningful, and 3 had a slightly worse THI score after treatments. As the study was uncontrolled placebo effects are not accounted for. It is worth noting too that all participants in the study had undergone between 3 and 8 previous treatments for tinnitus with medication, neurofeedback therapy, hearing devices, tinnitus masker devices, various other noninvasive forms of electrical stimulation, or cortical implantation.

A recent case report from the same research group compared the effects of VNS to the effects of sham stimulation in a 59-year-old man with refractory tinnitus [55]. It is not clear if the patient was one of the participants in the previous study. The methods used were the same as before with the exception that for 2 months (washout period) after paired VNS treatment the patient received sham stimulation (only tone presentation without the simultaneous electrical stimuli). After four weeks of VNS treatment the patient showed a reduction in THI scores of 48% and Tinnitus Reaction Questionnaire (TRQ [56]) scores by 68%. In contrast, sham stimulation led to a 27% increase in THI and 15% increase in TRQ scores. Again, VNS was observed to induce changes in the resting state oscillatory brain activity as measured with EEG. A significant decrease in relative power in the delta, theta, alpha, beta, and gamma frequency band was observed after VNS, suggestive of desynchronization; no changes were observed as a result of the sham stimulation.

It is worth noting that implantation of the vagal nerve stimulator is a full surgical procedure performed under general anaesthetic. This procedure is invasive and carries both short- and long-term risks. While authors stated that no major adverse events were observed in either study, several adverse effects of either the surgery or stimulation were listed, including infection of the extension lead requiring removal of the lead and electrode, inflammation at the abdominal surgical site, hoarseness during stimulation, transient left vocal cord hypomobility, and temporary increases in tinnitus symptoms. MicroTransponder Inc. have recently completed recruitment to a pilot study (NCT01962558) involving 30 participants randomized to receive either (1) VNS paired with tones or (2) VNS and unpaired tones hypothesised to be ineffective for tinnitus. The results of this study will be very informative for further clinical investigation.

### 3.5. Human Studies Using Transcutaneous VNS

An alternative noninvasive approach using transcutaneous stimulation of the vagus nerve (tVNS) is considered in parallel. Several studies examining the safety and efficacy of tVNS either alone or paired with acoustic stimulation have been conducted in recent years. Two consecutive reports from Kreutzer and colleagues [57, 58] describe the results of the single-arm pilot study looking at the tVNS without acoustic stimulation. The study was conducted in two phases, with different groups of participants with tinnitus in each phase. The first phase of the study was terminated early due to two cardiac events and was followed by detailed analysis of the ECG data collected throughout the study in all participants [57]. Authors concluded it was very unlikely that the events were caused by the tVNS device and the study progressed to phase 2. Analysis of the results from both phases showed that tVNS was suitable for long-term use, but without the use of paired acoustic stimuli it appeared to have no effect on tinnitus. Reductions in TQ scores were minimal (4 ± 8 in phase I and 3 ± 9 in phase II) and dropout was high (10 out of 23 patients in phase I and 6 out of 21 in phase II). This suggests that the treatment protocol, consisting of several hours of stimulation over 6 months, if effective at all might only be feasible for some patients. In addition, a number of adverse events were associated with the treatment including tingling sensation, dysesthesia, skin redness and pressure marks at the stimulation sites, painful stimulation, dyspnea of low intensity, headaches, hoarseness, nausea, and transient subjective hearing impairment. Overall it was concluded that tVNS was safe for prolonged use in patients without a history of cardiac disease.

Two studies have examined tVNS paired with acoustic stimulation in humans. Lehtimäki et al. [59] conducted a single-armed pilot study using music as their acoustic stimulus. The music was filtered of any frequencies that sounded like the individuals tinnitus. The treatment resulted in improvements in mood as measured with the WHO 5-point well-being questionnaire [60]. The mean well-being score increased from 56 points at baseline to 76 points after treatment. THI (severe at baseline) and mini-TQ questionnaire score decreased significantly, and subjective loudness and annoyance, measured with visual analogue scales (VAS), were also reduced after treatment. The auditory N1m response to the tinnitus frequency, measured by magnetoencephalography (MEG), was compared as “tVNS on” versus the “tVNS off” conditions; tVNS decreased the amplitude of the auditory N1m response, while the peak latencies did not change. Authors stressed the importance of these findings as a first demonstration of acute neuromodulative effects of tVNS on evoked auditory cortical responses. Safety of the tVNS was confirmed as no adverse events were reported.

Hyvärinen et al. [61] compared cortical oscillatory power (power spectral density, PSD) and synchronization (phase lag index, PLI) in people with moderately severe tinnitus and normally hearing controls using MEG. Specifically, baseline activity when the tVNS was not activated was compared between groups as well as differences in tone-evoked (tinnitus frequency in participants with tinnitus and 1 kHz in controls) brain activity between tVNS not activated and tVNS activated conditions in the tinnitus group. The results revealed higher PSDs in gamma band at baseline in tinnitus than in control group and higher PLIs particularly in beta and gamma bands in tinnitus patients compared to controls. tVNS had different effects on synchronicity in people with tinnitus and controls. In the tinnitus group, whole-head alpha activity, occipital alpha, and right temporal gamma activity increased during stimulation, and occipital theta activity decreased in response to tVNS. Hyvärinen et al. [61] also investigated the correlation of each measure with individual THI scores. Higher whole-head beta (*r* = 0.74), low temporal delta (*r* = −0.74), and low temporal theta (*r* = −0.71) were associated with higher THI scores when the tVNS was not activated. In addition, THI scores also correlated positively with the tVNS induced change in normalized PSD values for multiple frequency bands and brain regions. tVNS induced changes in synchrony correlated strongly with THI scores, at whole-head beta (*r* = −0.86), whole-head gamma (*r* = −0.95), and frontal gamma bands (*r* = −0.95). Overall, the picture is quite complicated and needs further investigation.

One study of tVNS for tinnitus is identified as ongoing. Li et al. [62] are conducting a randomized, single-blind, controlled clinical study (ChiCTR-TRC-14004940) comparing (1) tVNS only, (2) tVNS paired with sound stimuli, (3) nonvagus electrical stimulation of the outer ear, and (4) an electrical acupuncture group. They aim to recruit 120 participants and are using the THI as their primary outcome measure. As this questionnaire was also used in both previous studies of tVNS, the outcome of this study can be usefully compared or synthesised with existing data.

### 3.6. Chronic Electrical Vestibulocochlear Nerve Stimulation (VCNS)

An interesting alternative to the VNS is chronic electrical vestibulocochlear nerve stimulation (VCNS), where the stimulation electrode is surgically placed around the vestibulocochlear nerve and connected to a pulse generator placed under the skin [63]. In this approach the auditory pathways are stimulated directly. Bartels and colleagues [63] investigated safety and efficacy of this method in six participants with unilateral, severe, chronic refractory tinnitus and severe hearing loss in the tinnitus ear. Two participants dropped out, one due to the neurostimulation being unsuccessful after a period of three months and one due to other health and psychiatric problems not due to tinnitus, leaving four participants for long-term evaluation (42.5 months on average).

No side effects from VCNS were reported during long-term evaluation. Mean THI scores reduced from 77 points at the baseline to 55 points at 3 months and 38 points at long-term follow-up. This equated to a large clinical effect size (*d* = 1.75) at follow-up. For two participants, THI score was reduced after 3 months and for the other two participants THI scores were reduced after about 6 months of treatment. Self-rating of tinnitus severity was also reduced on a 10-point scale from a mean of 8 to 3 at long-term follow-up. As described by authors, while none of the participants' tinnitus disappeared during the treatment, “*all patients reported that the neurostimulation system transformed intrusive combination of noises into a single, pleasantly-perceived noise*” ([63] p.134).

No new studies of VCNS for tinnitus were identified as ongoing.

### 3.7. Brain Surface Stimulation

The earliest report of invasive electrical stimulation in tinnitus was provided by De Ridder et al. [64] who demonstrated suppression of tinnitus in a single patient following focal electrical stimulation of the primary auditory cortex. The patient, a 32-year-old woman, suffered from severe left-sided tinnitus following total loss of hearing in the left ear. Tinnitus was rated 9 on a 10-point VAS, and loudness was matched to an 80 dB external tone and between 8 and 9 kHz. Initially, TMS was applied to the right auditory cortex following functional magnetic resonance imaging (fMRI) to identify Heschl's gyrus. Tinnitus was completely abolished beyond the duration of stimulation. Subsequently, an extradural octopolar electrode was implanted onto the right auditory cortex for electrical stimulation via a neurostimulator which generated and delivered 0.5 msec pulses at a rate of 40 pps and 2.7 mA, to which the patient's tinnitus disappeared completely. However, three weeks after the operation, high-pitched tinnitus returned. The authors suggested this was due to cortical plasticity in response to constant stimulation at the high-frequency areas but could not explain how electrical stimulation of the cortex eliminated tinnitus.

Following their initial study, De Ridder et al. [18] examined the effect of implanted electrodes in a group of patients with moderate-to-severe tinnitus. Based on the similarity of tinnitus with pain, and the assumption that the sound is generated in the central nervous system, the authors argued that at least some forms of tinnitus are caused by neural plasticity and the reorganization of the central nervous system involving the auditory cortex. In addition, they assume that neural hyperactivity within the auditory system underlies the tinnitus abnormality. They thus propose that tinnitus can be treated by suppressing this hyperactivity by means of implanted electrodes in both the primary and secondary auditory cortices. They selected 12 participants who reported their tinnitus to be suppressed by at least 50% on a visual analogue scale by application of TMS. Primary and secondary auditory cortices were first localised using fMRI and frequency specific sounds (matched to tinnitus pitch), supposed to improve accuracy of finding the tinnitus “generator” in the auditory cortex. Two-pole electrodes were positioned such that one was intradural, on the primary auditory cortex, and the other was extradural, overlaying the secondary auditory cortex. The results were as follows: stimulation through the extradural electrode resulted in tinnitus suppression of 26% for patients with white noise tinnitus and 97% for those with pure tone tinnitus. As for the intradural electrodes, percentage suppression was not reported but only 2 patients failed to report an improvement in tinnitus, which suggests stimulation of primary auditory cortex provides more lasting suppression. Overall, patients with unilateral tinnitus reported better suppression than those with bilateral tinnitus. Moreover, in patients with white noise and pure tone tinnitus, electrical stimulation suppressed the pure tone tinnitus, while the white noise tinnitus remained unchanged. Importantly, tinnitus reappeared at some later time beyond the main study but it is not clear how long after the operation their tinnitus returned to its preoperative levels. The authors suggest that the intensity of the perceived tinnitus is related to the degree of cortical reorganization; however, this has since been shown not to be the case by a number of studies (e.g., see Sereda et al. [3]). Moreover, the assumption that tinnitus is generated within the auditory cortex, which guided the implantation loci, has not been established.

Seidman et al. [65] followed a similar procedure. Their reasoning was based on the thalamocortical dysrhythmia model [66, 67] which suggests that tinnitus emerges as a result of peripheral damage and deafferentation, leading to reduced neural firing rates in corresponding thalamocortical columns. Consequently, this leads to hyperactivity in the neighbouring regions (“edge effect”) and characteristic 40 Hz oscillations as the correlate of tinnitus. They therefore attempted to generate a therapeutic benefit by introducing an electrical signal in the vicinity of this reorganized auditory cortex. In two patients with chronic severe tinnitus, they first attempted to identify the tonotopic maps in Heschl's gyrus using MEG and fMRI, before implanting electrodes within areas showing increased activity. Electrode arrays were advanced into the brain tissue in Heschl's gyrus and in proximity to the area localised noninvasively. In one patient with bilateral, high-pitched tinnitus, loudness decreased significantly in both ears as the pulse generator was activated. In follow-up evaluations, this patient reported that tinnitus has remained significantly reduced (visual analogue scale rating). In the second patient with unilateral left-sided high-pitch narrow band tinnitus, neurostimulation was less successful. Several attempts at stimulation treatments over a period of two years did not diminish tinnitus. The reason for this discrepancy is not clear. The authors suggest that while electrical stimulation is clearly beneficial, perhaps in some patients areas such as the amygdala or hippocampus should be stimulated instead of the auditory cortex.

Most recently, De Ridder and Vanneste [68] report the case of a single tinnitus patient who experienced beneficial effect of an auditory cortex implant. In this study auditory cortex stimulation and C2 nerve stimulation were combined using implanted electrodes overlying the auditory cortex and subcutaneous electrodes modulating the C2 dermatome. It has been found that auditory deafferentation induces compensatory increases of somatosensory influences on the auditory pathways at the level of the dorsal cochlear nucleus [69] and that C2 electrical stimulation can suppress or enhance responses to sound via a pattern of inhibition and excitation of the principal cells in the ventral and dorsal division of the cochlear nucleus. It was hypothesised therefore that cross-modal convergence or interaction of multisensory neurons, whereby the modulation of activity evoked by one modality modulates the activity evoked by another [70], could be beneficial. The participant had suffered from severe unilateral right-sided tinnitus for 1.5 years prior to testing. Subjective tinnitus loudness score was 8.5/10 on a 10-point scale, matched to a 6 kHz with an audiometric threshold that also dipped at 6 kHz. The patient had no sign of somatosensory modulation of tinnitus. fMRI was performed to identify the auditory cortex and subsequently an electrode was implanted overlying the secondary auditory cortex. The authors report a complete suppression of the pure tone component of the tinnitus; however, the noise-like component was only suppressed to 4 on a 10-point scale, which worsened again. Subsequently, transcutaneous electrical nerve stimulation (TENS) at C2 reduced the noise from 7 to 1-2/10 consistently over 4 weekly sessions. The patient used the TENS on a daily basis with good result but after 3 months the effect diminished. A permanent subcutaneous electrode was then inserted which activated the auditory cortex electrode. As a result, noise-like tinnitus reduced to 4/10 and remained so for 5 years, while pure tone tinnitus remained absent. Despite this, the exact mechanism involved in the C2 stimulation for tinnitus relief remains unknown. Moreover, given that only a single patient was examined, it is not possible to speculate on its general effectiveness. The method requires application in a larger group of patients as it is possible that patients with different tinnitus characteristics will respond differently to this form of treatment.

Based on the similarity of pain and tinnitus pathways, ACC and anterior insula have been suggested to be a core network for the noise cancelling system. Fluctuations of activity in the ACC and anterior insula determine whether a near threshold pain stimulus is consciously perceived or not [71]. A number of anatomical and functional studies indicate that tinnitus distress may be related to a cortical network involving the ACC, anterior insula, and the parahippocampal area [72, 73]. In particular, self-reported tinnitus distress may be related to increased activity in the dorsal ACC and insula [40]. De Ridder et al. [74] report the results of a study on two patients who underwent implantation of electrodes in the dorsal ACC. Both patients suffered from chronic tinnitus subjectively rated 10/10 on a scale for loudness and highly on questionnaire measures of distress. Initially, fMRI was used to identify target zones for neurostimulation using sound stimuli matched to the tinnitus frequency. TMS was applied to various regions including the auditory cortices and the prefrontal regions over repeated sessions, but this did not produce lasting benefits to either patient. Subsequently, both patients underwent surgery for implantation of electrodes in the dorsal ACC. One patient reported dramatic improvement in both tinnitus severity and loudness after one week, which halved from the preoperative stage. Additional stimulation after four weeks resulted in further reduction of tinnitus loudness and distress ratings. These effects had remained two years after implantation. Conversely, the second patient did not report any change as a result of the implant which was trialled at various stimulation configurations. The authors found increased functional connectivity between dorsal ACC, pregenual ACC, parahippocampal area, and auditory cortex in the patient who reported improvements in their tinnitus. The patient who reported no change demonstrated decreased functional connectivity in this network. Clearly, while the results of this study offer possibilities for tinnitus relief, the hypotheses need further evaluation with a wider pool of tinnitus patients, particularly with regard to assessment of connectivity. Whether the assessment of functional connectivity in the specified network is predictive of postoperative success cannot be ascertained from this.

It is clear that not all patients with tinnitus benefit from neurostimulation via implanted electrodes. De Ridder and Vanneste [68] have shown that functional connectivity analysis based on EEG resting state activity between the auditory cortex and parahippocampus may determine whether a tinnitus patient will respond to a cortical implant. This might be a necessary initial step prior to neurosurgery given the various risks attached with the latter.

### 3.8. Deep Brain Stimulation

Deep brain stimulation (DBS) involves the implantation of a neurostimulator within the brain to deliver electrical pulses. It has been used with varying degrees of success to treat a number of neurological disorders, most notably movement and affective disorders such as Parkinson's disease [75] but also pain [76]. For tinnitus, DBS aims to disrupt the communication between various parts of the brain involved by modifying or preventing the abnormal neural signal from reaching the auditory cortex. A number of studies have assessed the therapeutic merits of DBS to affect mainly subcortical (but also cortical) structures which are thought to be involved in tinnitus including the thalamus and the inferior cortex.

The first DBS study in tinnitus was a case series reported by Shi et al. [77]. They collected self-rating and psychoacoustic measures of tinnitus loudness in seven patients who were implanted for movement disorders but also reported having tinnitus. Electrodes were placed unilaterally or bilaterally in the ventral intermedius nucleus of the thalamus for control of involuntary tremors. Three of the seven patients reported quieter tinnitus when DBS was turned on. Decreases in matched tinnitus loudness were found in two patients exceeding the range of normal test-retest variations (±2 dB), which agreed with their subjective rating of their DBS-related tinnitus changes. Therefore, a placebo effect was ruled out by the authors. For the two tested patients whose tinnitus responded to DBS, tinnitus remained suppressed for 15 to 20 minutes after stimulation was turned off. None of the patients reported stimulation-related changes in hearing and audiometry confirmed there were no changes in hearing thresholds. The results indicate that changes in activity within nonauditory system may be involved in the maintenance of tinnitus. The ventral medial nucleus is involved in motor functions and mainly receives input from the cerebellum and the globus pallidus. It is the site of DBS for motor-related movements such as multiple sclerosis [78] and Parkinson's disease [79]. However, it is not clear why stimulation of motor centres affects the auditory system, and given that hearing thresholds were not affected, it is unlikely that the stimulation spreads to other areas of the thalamus that comprise the auditory pathways. In the two patients who did not respond to stimulation, tinnitus had existed for more than 20 years, compared to less than 10 years in responders, which the authors suggest possibly indicating “decreased plasticity in factors related to tinnitus perception over very long periods of time” ([77] p.286).

In another study, Cheung and Larson [80] applied DBS to a locus of caudate neurons (area LC), a subsite of striate nucleus and a part of basal ganglia. The caudate nucleus also receives input from the auditory cortices. It was assumed that stimulation of the basal ganglia would disrupt attention to auditory cortical representations of tinnitus. Six patients with chronic tinnitus were recruited who were primarily being treated for Parkinson's disease or tremors. Five patients underwent electrical stimulation of the caudate. In one patient, tinnitus was completely abolished by stimulation. In the remaining participants, tinnitus was decreased to varying extents in both ears. Three patients had a preoperative and postoperative audiogram; none showed any significant change postoperatively. Overall, the results indicated that LC neuromodulation by DBS can decrease tinnitus loudness. The authors speculate that while the LC is not part of the classical auditory pathway, it may play a role in integration of phantom sensations generated from the central auditory system with areas involved in perceptual awareness.

The inferior colliculus (IC) and its various subdivisions are a major relay centre for auditory signals arriving from the cochlear nucleus. It is situated within the midbrain and plays a significant role in auditory signal integration and frequency and pitch processing. Using fMRI, Melcher et al. [81] found abnormal activity in IC of patients with tinnitus. In order to assess the potential effects of IC stimulation on tinnitus, Offutt et al. [82] compared the extent of suppression and facilitation elicited in the central IC by different stimulation paradigms. Using a guinea pig model, they found that stimulation of dorsal IC induces both suppressive and facilitatory changes across the central IC which can occur during stimulation and that these remain after stimulation. Interestingly, stimulation of dorsal IC induced greater suppression when paired with broadband noise stimulation. The study, while not using tinnitus induced animals, demonstrates that stimulation of the dorsal IC can induce plasticity within the central IC and concomitant changes in activity, which may be applicable in treating tinnitus. Recently, Shekhawat et al. [23] showed that a combination of noise and cortical stimulation using TMS may be more effective in suppressing tinnitus than either one alone. The study by Offutt and colleagues can guide future TMS studies to target the appropriate regions of the brain for more optimal therapeutic results.

It must be noted that the studies attempting DBS are principally aimed at alleviating other coexisting conditions (Parkinson's disease, tremor). Therefore, the interacting effects of these comorbidities with tinnitus are not known. Furthermore, a comprehensive explanation of this neuromodulation is not presented. It is not clear how the minute effects of weak currents exert an effect on isolated neuron membrane potentials and lead to significant changes in network dynamics at the cellular (e.g., timing) and/or network levels. It is also not clear what frequency of oscillations and intensities are necessary to induce neural changes that improve tinnitus therapy in patients with differing clinical manifestations. Such a framework for neuromodulation is currently lacking. Moreover, it is intriguing that, in most of the reported studies, hearing function (audiometric thresholds) is not affected given that tinnitus is an auditory sensation that primarily involves the auditory system. Therefore, any changes in the tinnitus perception would be expected to also affect other aspects of audition; but none are reported in these studies.

One relevant study was identified as ongoing. van den Berge and van Dijk are planning a safety/efficacy study (NCT02630589) involving an auditory brainstem implant for tinnitus. Ten participants with severe intractable tinnitus will be neurosurgically implanted, whereby an electrode array is inserted into the lateral recess of the fourth ventricle and placed on the cochlear nucleus. This study will add to the slowly growing body of evidence for the use of invasive techniques to treat intractable tinnitus.

## 4. Conclusions

Each tinnitus patient represents a unique combination of tinnitus characteristics, aetiology, pathophysiology, and psychoperceptual factors. Therefore, it is unlikely that any treatment will provide relief for all tinnitus sufferers, as indeed is demonstrated by the results of the studies reviewed here. While their precise mechanism may not be known, noninvasive methods are worth exploring as they seem to provide comparable benefits to invasive methods, although this will not be the case for every individual patient. It is possible that structures involved in neuromodulation are not involved in generation and maintenance of tinnitus in all but only a subtype of patients. Therefore, neuromodulation offers another venue for identifying the subtypes of tinnitus, which is the goal of a current European-funded Cooperation in Science and Technology (COST Action) program for a Tinnitus Research Network (TINNET; http://tinnet.tinnitusresearch.net/index.php/2015-10-29-10-22-16/wg-3-neuroimaging) to identify subtypes of tinnitus and their neural correlates and thus develop innovative hypothesis driven treatment approaches.

## Figures and Tables

**Table 1:** Summary of study findings.

Intervention	Reference	Study design	Physiological effect	Clinical effect	Adverse effect	Notes on findings
Noninvasive electrical stimulation of the peripheral organs	Lee et al., 2014 [38]	Randomized controlled trial.	Not assessed.	THI scores decreased from 49 to 43 in the treatment group with no change in controls. TENS seemed to be more effective in people with low frequency tinnitus and milder hearing losses.	Mild side effects in eight patients: 4 had dizziness, 3 had headache, and 1 had facial numbness. Side effects disappeared after cessation of treatment.	Although statistically significant the 6.6-point decrease in THI score is not clinically meaningful.
	Mielczarek et al., 2013 [36]	Nonrandomized controlled study.	Not assessed.	No clinical measures.	Pain/discomfort during stimulation, which was resolved by reducing intensity of the current.	Self-reported presence of tinnitus halved in both groups after treatment.
	Mielczarek and Olszewski, 2014 [37]	Double-blind placebo-controlled trial.	Not assessed.	No clinical measures.	No harmful effects were observed.	Self-reported presence of tinnitus halved in the treatment group but was only slightly reduced in placebo.

Vagus nerve stimulation	De Ridder et al., 2014 [53]	Uncontrolled before and after study, pilot/phase I/safety and efficacy.	VNS decreased band power in the delta (1–4 Hz) and theta (4–8 Hz) bands in subjects who responded to therapy and increased band power in those bands in subjects who did not respond to the therapy. The average difference in band power change between VNS and sham trials in participants with tinnitus was strongly correlated with the THI for both delta (*r* = 0.83) and theta (*r* = 0.74) bands.	For the whole group the average decrease in THI score from the baseline was 11% immediately after the treatment and 12% for the follow-up in comparison with the baseline and therefore was not clinically significant.	Patient 001: redness of the abdominal site and vocal cord hypomobility after the surgery; resolved within 2 weeks. Patient 003: infection (lead and electrode explanted). Patient 004: increased tinnitus symptoms for the first week, ongoing depression requiring medication, hoarseness during stimulation, difficulty tolerating standard settings, depressive episode, and failed suicide attempt.	Post hoc analysis of “drug-taking” versus “non-drug-taking” group: 3 of 5 patients in the nondrug group had clinically meaningful decrease in the THI. Only one patient had improvement of 20 points or more.
	De Ridder et al., 2015 [55]	Case study. Cross over- paired VNS followed by sham.	EEG: resting state, a significant decrease of relative power in the delta, theta, alpha, beta, and gamma frequency band effects after VNS, suggestive of desynchronization. No difference after sham stimulation.	At 4 weeks: THI reduced by 48% and TRQ reduced by 68%. 14-point reduction on the THQ and 23-point reduction on the TAQ. Sham stimulation: increase in THI of 27% and TRQ of 15%. THQ increase 3% and TAQ increase 9%.	Hoarseness during stimulation, transient left vocal cord hypomobility, and slight inflammation at the abdominal surgical site (last two resolved after 2 weeks). Slight worsening of tinnitus after sham treatment.	Reduction in all bands: usually lower bands go down and alpha goes up, contradictory to other studies.

Transcutaneous vagus nerve stimulation (tVNS)	Kreuzer et al., 2012 [57]	Pilot study, retrospective. Feasibility and safety study with an open, single-armed study design.	Not assessed.	Not assessed.	Two adverse cardiac events (one severe) were registered but considered very unlikely to have been caused by the tVNS device. Also reported were headache, breathing difficulties, chest sensation, dizziness, subjective hearing impairment, worsening of tinnitus, neck pain, croakiness, and sleeping disorder.	Nevertheless, in the light of the potential of tVNS to modulate conduction system of the heart, ECG recordings are recommended in every study of tVNS in order to obtain further safety data.
	Kreuzer et al., 2014 [58]	Single-arm open-label pilot study.	Not assessed.	Baseline to 24-week reduction in TQ score of 2 points; not significant in either group.	Two cardiac events. Adverse events: tingling sensation, dysesthesia, skin redness and pressure marks at the stimulation sites, painful stimulation, dyspnea of low intensity, and headaches. Chest pain, voice alteration/hoarseness, nausea, arrhythmia, dizziness, transient subjective hearing impairment, neck pain, and numbness.	High dropout rate suggests that a therapeutic tVNS application of several hours per day over a period of 6 months is only feasible for some patients. There was no clinically relevant improvement of tinnitus complaints. Data suggest tVNS to be considered safe in patients without a history of cardiac disease.
	Hyvärinen al., 2015 [61]	Prospective, control group and tinnitus group, comparisons both between control and TI and within TI and stimulation versus no stimulation.	Tinnitus patients differed from controls in the baseline condition (no tVNS applied), measured by both cortical oscillatory power and synchronization, particularly in beta and gamma bands. tVNS induced changes in synchrony.	Changes in synchrony correlated with THI scores, at whole-head beta (*r* = −0.857), whole-head gamma (*r* = −0.952), and frontal gamma bands (*r* = −0.952).	None reported.	
	Lehtimäki et al., 2013 [59]	Pilot study, short-term therapeutic trial, before and after study.	Auditory N1m responses: “tVNS on” versus “tVNS off” conditions. tVNS decreased amplitude of the auditory N1m response. Peak latencies remained unchanged.	tVNS plus sound therapy led to improved well-being; WHO 5-point well-being questionnaire mean scores increased from 56 to 76. Mean THI and mini-TQ questionnaires scores decreased significantly.	None reported.	The most significant finding of this study was the MEG demonstration of acute neuromodulative effects of tVNS on evoked auditory cortical responses.

Vestibulocochlear nerve stimulation	Bartels et al., 2007 [63]	Before and after study with long-term follow-up. No control group.	Not assessed.	Mean THI score reduced from 77 at baseline to 55 at 3 months and 38 points at 42.5 months.	None reported.	All four patients reported that treatment changes their tinnitus sound from an intrusive combination of noises into a single, pleasantly perceived noise.

Transcranial alternating current stimulation (tACS)	Vanneste et al., 2013 [40]	Randomized trial comparing three interventions.	Not assessed.	Numeric rating scales only.	None reported.	There were no significant improvements in the tACS group. A large transient suppressive effect was noted for one comparison group receiving transcranial random noise stimulation.
	Vanneste et al., 2013 [41]	Randomized placebo-controlled trial of tACS.	No significant EEG power changes after tACS.	Numeric rating scales only.	None reported.	tACS did not improve rating of tinnitus annoyance or loudness.
	Claes et al., 2014 [42]	Retrospective analysis of clinical data from patients receiving tACS or random noise stimulation.	Not assessed.	Numeric rating scales only.	None reported.	Small statistically significant improvements in ratings noted for tRNS group only.

Brain surface stimulation	De Ridder et al., 2004 [64]	Surgical case report, extradural electrical stimulation.	Not assessed.	No clinical measures.	None reported.	Tinnitus initially suppressed for three weeks after implantation with continuous stimulation which thereafter required adjustment.
	De Ridder et al., 2006 [18]	Cohort study, primary and secondary auditory cortex electrodes.	NA.	Visual analogue scales only.	Some reports of dizziness, altered spatial localisation, and altered hearing. Epileptic seizure in 2 patients after prolonged stimulation with an external stimulator.	Self-reported improvements were noted for a subgroup of patients who had pure tone tinnitus.
	Seidman et al., 2008 [65]	Two case reports, electrode arrays advanced into the brain tissue in Heschl's gyrus.	NA.	Patient 1: clinically significant improvement in THI score 17 days after implantation. Patient 2: particle suppression of tinnitus only.	None reported.	Patient 1 experienced long-term improvements, whereas patient 2 reported tinnitus returning to original level 2 years after implantation.
	De Ridder et al., 2016 [74]	Two case reports, anterior cingulate implants.	Not assessed.	Tinnitus questionnaire not reported postoperatively.	One implant removed after 2 years due to possible infection.	Partial suppression of tinnitus in one patient only. Tinnitus was unchanged in patient 2.

Deep brain stimulation	Torres et al., 2010 [78]	Cohort study, ventral intermedius nucleus of the thalamus implant.	Not assessed.	Numeric rating scales only.	None reported.	Decreased tinnitus loudness during stimulation reported by 2 of 7 patients and for 15–20 minutes after stimulation was switched off.
	Cheung and Larson, 2010 [80]	Pilot study, locus of caudate neurons implant.	Not assessed.	Numeric rating scales only.	Increased loudness in one ear reported by one patient (suggested likely micro-lesion effect).	Tinnitus loudness was acutely reduced in all patients where the electrode traversed the locus of caudate neurons (5 out of 6).
